# *Peganum* spp.: A Comprehensive Review on Bioactivities and Health-Enhancing Effects and Their Potential for the Formulation of Functional Foods and Pharmaceutical Drugs

**DOI:** 10.1155/2021/5900422

**Published:** 2021-06-27

**Authors:** Javad Sharifi-Rad, Cristina Quispe, Jesús Herrera-Bravo, Prabhakar Semwal, Sakshi Painuli, Beraat Özçelik, Furkan Ediz Hacıhasanoğlu, Shabnum Shaheen, Surjit Sen, Krishnendu Acharya, Marjan Amirian, Carla Marina Salgado Castillo, María Dolores López, Mauricio Schoebitz, Miquel Martorell, Tamar Goloshvili, Ahmed Al-Harrasi, Ahmed Al-Rawahi, Manoj Kumar, Hafiz Ansar Rasul Suleria, William C. Cho

**Affiliations:** ^1^Phytochemistry Research Center, Shahid Beheshti University of Medical Sciences, Tehran, Iran; ^2^Facultad de Medicina, Universidad del Azuay, 14-008 Cuenca, Ecuador; ^3^Facultad de Ciencias de la Salud, Universidad Arturo Prat, Avda. Arturo Prat 2120, Iquique 1110939, Chile; ^4^Departamento de Ciencias Básicas, Facultad de Ciencias, Universidad Santo Tomas, Chile; ^5^Center of Molecular Biology and Pharmacogenetics, Scientific and Technological Bioresource Nucleus, Universidad de La Frontera, Temuco 4811230, Chile; ^6^Department of Biotechnology, Graphic Era University, Dehradun, 248001 Uttarakhand, India; ^7^Uttarakhand State Council for Science and Technology, Vigyan Dham, Dehradun, 248007 Uttarakhand, India; ^8^Himalayan Environmental Studies and Conservation Organization, Prem Nagar, Dehradun, 248001 Uttarakhand, India; ^9^Department of Food Engineering, Faculty of Chemical and Metallurgical Engineering, Istanbul Technical University, Maslak, TR-34469 Istanbul, Turkey; ^10^BIOACTIVE Research & Innovation Food Manufacturing Industry Trade Co. Ltd., Katar Street, Teknokent ARI-3, B110, Sarıyer, 4467 Istanbul, Turkey; ^11^Department of Plant Sciences, LCWU, Lahore 54000, Pakistan; ^12^Molecular and Applied Mycology and Plant Pathology Laboratory, Department of Botany, University of Calcutta, Kolkata 700019, India; ^13^Department of Botany, Fakir Chand College, Diamond Harbour, West Bengal 743331, India; ^14^Department of Plant Production, Faculty of Agronomy, Universidad de Concepción, Avenida Vicente Mendez, 595, Chillán 3812120, Chile; ^15^Departamento de Suelos y Recursos Naturales, Facultad de Agronomía, Universidad de Concepción, Concepción, Chile; ^16^Department of Nutrition and Dietetics, Faculty of Pharmacy and Center for Healthy Living, University of Concepción, 4070386 Concepción, Chile; ^17^Institute of Botany, Department of Plant Physiology and Genetic Resources, Ilia State University, Tbilisi, Georgia; ^18^Natural and Medical Sciences Research Centre, University of Nizwa, Birkat Al Mouz 616, Oman; ^19^Chemical and Biochemical Processing Division, ICAR-Central Institute for Research on Cotton Technology, Mumbai 400019, India; ^20^Department of Agriculture and Food Systems, The University of Melbourne, Melbourne 3010, Australia; ^21^Department of Clinical Oncology, Queen Elizabeth Hospital, Kowloon, Hong Kong

## Abstract

The genus *Peganum* includes four species widely distributed in warm temperate to subtropical regions from the Mediterranean to Mongolia as well as certain regions in America. Among these species, *Peganum harmala* L., distributed from the Mediterranean region to Central Asia, has been studied and its phytochemical profile, traditional folk use, and application in pharmacological and clinical trials are well known. The review is aimed at presenting an insight into the botanical features and geographical distribution of *Peganum* spp. along with traditional folk uses. This manuscript also reviews the phytochemical profile of *Peganum* spp. and its correlation with biological activities evidenced by the *in vitro* and *in vivo* investigations. Moreover, this review gives us an understanding of the bioactive compounds from *Peganum* as health promoters followed by the safety and adverse effects on human health. In relation to their multipurpose therapeutic properties, various parts of this plant such as seeds, bark, and roots present bioactive compounds promoting health benefits. An updated search (until December 2020) was carried out in databases such as PubMed and ScienceDirect. Chemical studies have presented beta-carboline alkaloids as the most active constituents, with harmalol, harmaline, and harmine being the latest and most studied among these naturally occurring alkaloids. The *Peganum* spp. extracts have shown neuroprotective, anticancer, antimicrobial, and antiviral effects. The extracts are also found effective in improving respiratory disorders (asthma and cough conditions), dermatoses, and knee osteoarthritis. Bioactivities and health-enhancing effects of *Peganum* spp. make it a potential candidate for the formulation of functional foods and pharmaceutical drugs. Nevertheless, adverse effects of this plant have also been described, and therefore new bioproducts need to be studied in depth. In fact, the design of new formulations and nanoformulations to control the release of active compounds will be necessary to achieve successful pharmacological and therapeutic treatments.

## 1. Introduction

The genus *Peganum* is a perennial, herbaceous, glabrous to pubescent, and wild flowering plant with short creeping roots and belongs to the family Zygophyllaceae [1–3]. The genus has four main species (*Peganum harmala* L., *Peganum mexicanum* Gray, *Peganum nigellastrum* Bunge, and *Peganum multisectum* (Maxim.) Bobrov) having significance in health promotion and various biological activities in the human body. These species are widely distributed in warm temperate to subtropical regions from the Mediterranean to Mongolia of the Old World and from Texas to Mexico in the New World [4–10] (Table 1). Among the different species of *Peganum*, *P. harmala* has been studied well in relation to its application as a traditional folk medicine to modern pharmacological usage. *Peganum* spp. has been employed in the treatment of diabetes, rheumatism, Parkinson's disease, hypertension, jaundice, and asthma. The plant is also widely used in traditional Chinese medicine for the treatment of apoplexy and lumbago and also as a stimulant for improving the function of the central nervous system [11]. The benefits of these species are associated with its phytochemical profile. The seeds, roots, leaves, fruits, stems, and flowers of *Peganum* spp. have been widely studied for their phytoconstituents. Bioactive alkaloids (quinazoline alkaloids and *β*-carboline), essential oil components, and other phenolic compounds mainly contribute to the health-promoting effects. Harmaline, harmine, harmol, harmalol, vasicine, vasicinone, deoxyvasicine, and deoxyvasicinone are few examples of bioactive alkaloids present in *Peganum* spp. Bioactive compounds are important for their numerous biological functions, viz., anticancer, antidiabetic, antimicrobial, anti-inflammatory, antiviral, antidepressant, and antioxidant [12]. These biological activities have been well documented via *in vivo* and *in vitro* investigations.

**Figure 1:**
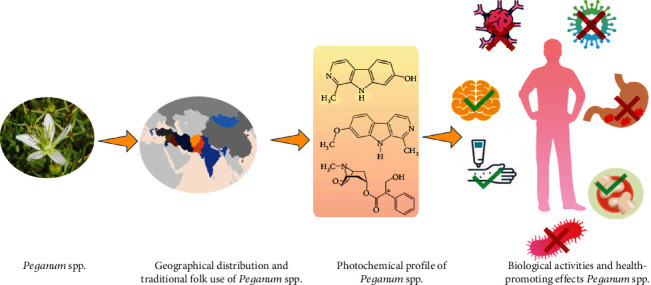
Various components discussed in the review article.

This current review is aimed at critically discussing the botanical features and geographical distribution of *Peganum* spp. along with traditional folk uses. It highlights the phytochemical profile of the *Peganum* genus along with the biological activities proven by *in vivo* and *in vitro* trials. Finally, the health-promoting effects of *Peganum* spp. plant extracts are well established in this review. The content discussed in this manuscript has been summarized in Figure 1.

## 2. Geographical Distribution of *Peganum* spp. and Botanical Features

**Figure 2:**
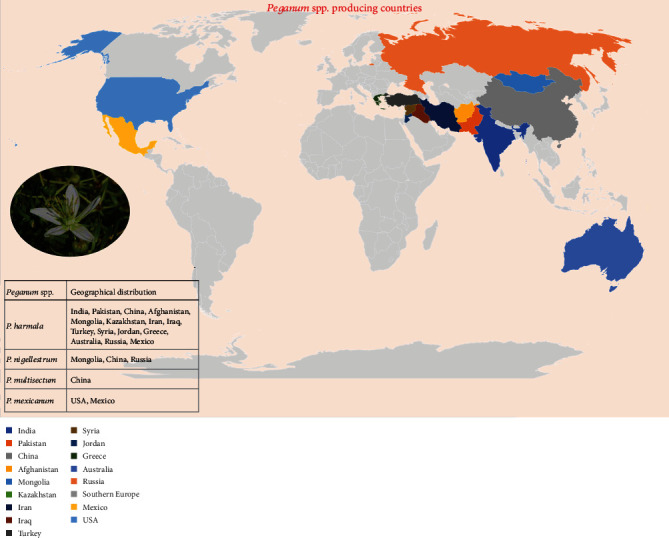
Map presenting the distribution pattern of *Peganum* spp. throughout the globe.

*P. harmala* is among the most studied species. Commonly known as “Harmal” or “Suryin Rue”, it is distributed from the Mediterranean region to Central Asia. It is a drought-tolerant plant, and in Central Asia, this species is found in Mongolia, Kashgaria, Tsaidam, Dzungaria, and Tibet [13]. This species is native to arid and semiarid regions, is widely distributed in North Africa, and is also found in the Middle East, Turkey, Pakistan, India, Iran, Kazakhstan, Mexico, South America, and many other countries [6, 14–21]. *P. nigellastrum* and *P. multisectum* are two other species which are gaining importance due to their health-promoting effects. These species are grown in northwestern China generally in arid and semiarid regions, including Xinjiang Province, Mongolia, and Russia and are vital components of desert vegetation [8, 22–25]. *P. mexicanum* is commonly found growing in the United States and Mexico of North America [23]. A distribution pattern of the *Peganum* spp. throughout the globe is presented in Figure 2.

As already mentioned, the genus *Peganum* is a perennial, herbaceous, glabrous to pubescent, and wild flowering plant with short, creeping roots. Leaves alternate, entire or multifide to palmatisected; flowers 1–3, on subterminal leaf opposed peduncles, white; sepals 4–5, entire to lobed; petals 4–5, whitish to yellow, imbricate, oblong; stamens 8–15, anther bicelled, filamentous inserted at the base of the disc; ovary 2–4 locular, globular; ovules many in each chamber; fruits capsule, globular in shape, splitting by 3 valves or indeniscent fleshy; seeds many, blunt-top and sharp-top shape, testa rough, spongy; curved embryo [26]. Morphological distinctive features of the main three species *P. harmala*, *P. multisectum*, and *P. nigellestrum* are presented in Table 1.

## 3. Traditional Uses

*P. harmala* has been used in various therapeutic, superstitious, and even ritual applications in different cultures and ethnicities. One of the superstitious uses of *Peganum* is found in Morocco where the plant is set on fire for the protection against ghosts and goblins. In Iran, Turkey, China, and other Arab countries in Africa and Asia, it is used to protect a person or condition from the evil eye [29]. One the rituals around the world related to *Peganum* that should be mentioned is a special ceremony in Zoroastrianism, where a prayer is recited and the *Peganum* is set on fire; the other is where *Peganum* is burned in a wedding ceremony in India, so that the bride and groom can avoid a life of darkness [30]. However, the medical aspects of *Peganum* are more prominent as they have long been used in the treatment of diabetes, asthma, arthritis, hypertension, and many other diseases in different cultures. A detailed list of the various traditional uses of the *Peganum* spp. is displayed in Table 2.

## 4. Essential Oils and Phytochemical Composition of *Peganum* Genus

### 4.1. Essential Oil and Its Components

**Figure 3:**
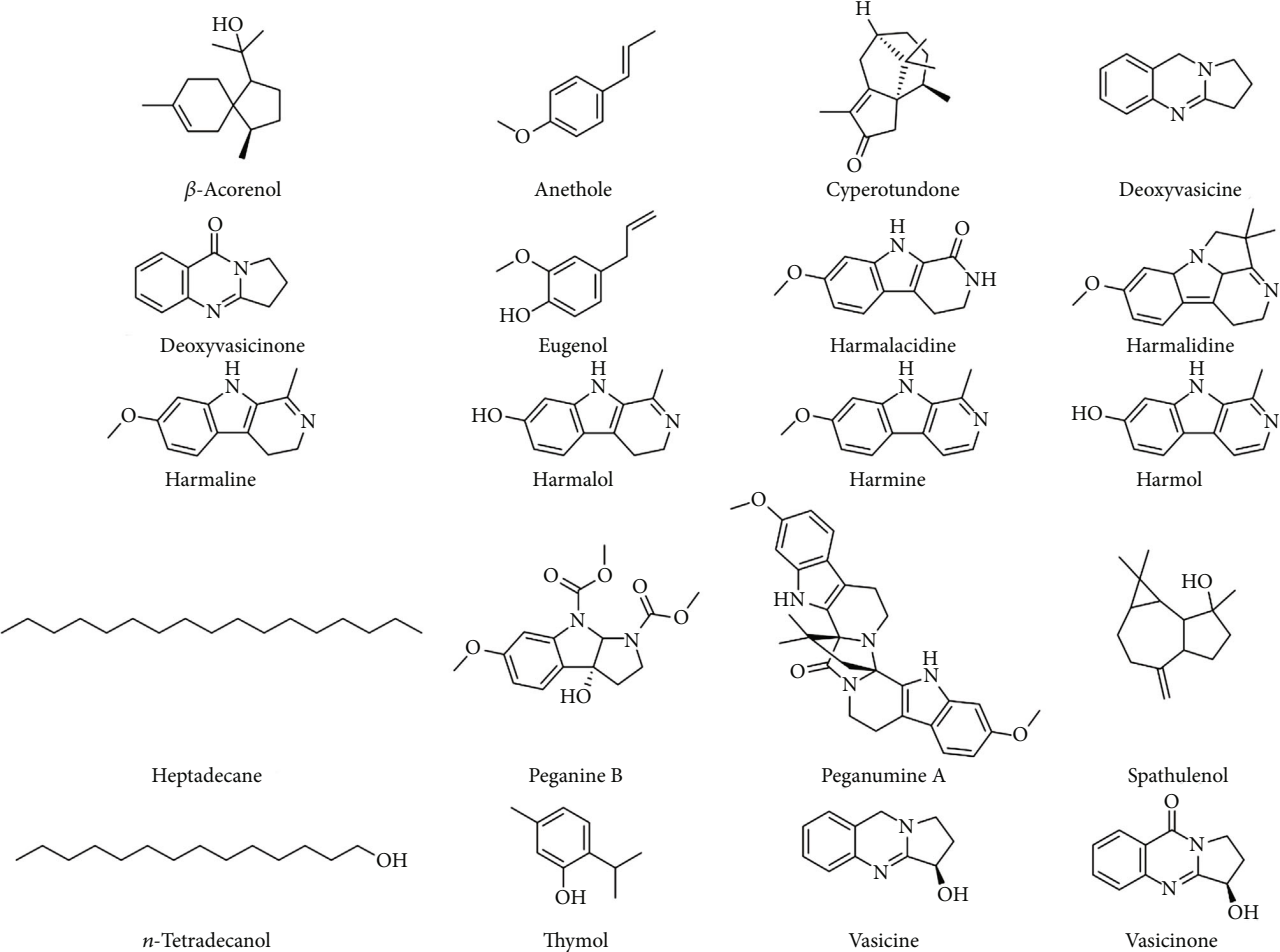
Phytochemical compounds isolated from different *Peganum* spp.

Essential oils are volatile, complex, and natural compounds produced by aromatic plants as secondary metabolites [38]. They are used in different industries including aromatherapy, fragrance and flavor industry, biotechnology and pharmaceutical industry, cosmetic and soap industry, and agriculture industry. The chemical composition of essential oils from the *Peganum* genus has been reported by a limited number of authors. Only a few studies are available in the literature about the essential oil composition of *P. harmala* from Morocco [37, 39], Algeria [37], Iran [40], Saudi Arabia [41], Tunisia, Libya, and Egypt [37]. The exhaustive profile of essential oil components from *Peganum* spp. is displayed in Table 3. Oxygenated monoterpenes, sesquiterpenes, eugenol, and oxygenated sesquiterpenes are the principal components in the essential oils from the populations of Morocco, Algeria, Tunisia, Libya, and Egypt, respectively.  Figure 3 summarizes some of the most important phytochemical compounds isolated from *Peganum* spp. from different regions.

### 4.2. Other Biologically Active Phytochemicals

The chemical diversity and biologically active compounds in different parts of *P. harmala* and other species of the genus *Peganum* has been reported by the several authors around the world (Table 4).

## 5. Biological Activities

### 5.1. *In Vitro* Studies

#### 5.1.1. Anticancer Activity

*P. harmala* has been demonstrated to inhibit the effect on the progress of some type of cancers by reducing the growth rate of cancerous cells. The use of Annexin-V-fluos for apoptosis analysis of MDA-MB-231 breast cancer cells showed that the seed extract of *P. harmala* led to declining growth rates depending on the concentration and time that the cells were exposed. A concentration of 30 *μ*g/mL not only reduced the growth rate but also subjected 50% of the cells to apoptosis in nearly 24 h. Furthermore, it was also noticed that the *P. harmala* seed extract caused an extrinsic pathway-induced apoptosis of the cells [88]. Deoxypeganine, harmine, and peganine compounds were tested as a multidrug resistance inhibitor of cancer cells, but they were not successful against the MDR1 gene-transfected mouse lymphoma cell line, even when the highest dose of 40 *μ*g/mL was used [89]. The alkaloid fraction of *P. harmala* seeds was tested for cell toxicity against UCP-Med and Med-mek carcinoma and UCP-Med sarcoma (applied at concentration levels of 20, 40, 60, 80, and 100 *μ*g/mL). Application of the alkaloid fraction of *P. harmala* to UCP-Med carcinoma resulted in extremely slow proliferation of the cells in the first 24 h, followed by cell lysis that started between incubation of 24 and 72 h as per the variation in the treatment. Against the UCP-Med sarcoma, only 20 *μ*g/mL of treatment was enough to slow down the proliferation in the first 24 h. After 24 h exposure of 60–100 *μ*g/mL, the cell death rate was 100%. When alkaloids were applied to Med-Mek carcinoma, a slower growth was observed compared to the other cell lines. Moreover, the inhibition of growth was instantly detected upon exposure to alkaloids, specifically at 80 and 100 *μ*g/mL concentrations. Additionally, the alkaloid fraction of *P. harmala* created cytotoxic effects on the tumoral cell lines, directly proportional with the time and concentration of exposure [90].

Cytotoxic effects of raw alkaloid extracts of *P. harmala*'s fruits (TAFr), seeds (TASe), roots [70], and aerial parts (TAAp) were evaluated by MTT (3-(4,5-dimethylthiazol-2-yl)-2,5-diphenyltetrazolium bromide) assay and quantitative video microscopy analysis of cancer cells (Hs683 and B16F10; the resistant ones to proapoptotic stimuli A549, U373, MCF7, SKMEL-28). Cytotoxic effects were observed on all cancer cells after 72 h of treatment with an IC_50_ range between 1 and 52 *μ*g/mL. Extracts of *P. harmala* presented a clear inhibition of all cancer cell lines, but SKMEL-28 had 38% of survivability after treatment with TAAp at the highest tested dose (100 *μ*g/mL). Analysis of A549 lung cancer cells exhibited only TAAp showing cytotoxic effects instead of cytostatic effects. However, TAFr, TASe, and TAR showed cytostatic effects. While the global growth rate values of TAAp, TASe, and TAFr were 0.19, 0.3, and 0.26, that of TAR was 0.62. Based on cell death rates, TAFr, TASe, and TAR inhibited cancer growth, but not induced cell death. In spite of that, TAAp caused cell death in the same cells by 0.29 [91].

#### 5.1.2. Neuroprotective Activity

Acetylcholinesterase inhibitors (AChEIs) are used for the treatment of Alzheimer's disease. A study which used thin-layer chromatography (TLC, bioautographic assay) showed that methanol extracts of the *P. nigellastrum* seed had an important inhibitory effect. Only 0.01 *μ*g of harmine, harmaline, harmol, and galathamine (which are known as AChE inhibitors) inhibited AChE. Also, 0.1 *μ*g concentrations of vasicine and harmol were enough to inhibit the AChE activity. Not only that, vasicinone, deoxyvasicinone, deoxyvasicine, and nigellastrines I and II exhibited low AChEI effect (1 *μ*g). Therefore, *P. nigellastrum* alkaloids presented a high potential to be used as an AChE inhibitor [66]. The harmane alkaloid from *Peganum* proved to have the most powerful AChE inhibitor activity although butyrylcholinesterase (BChE) inhibitor activity was lower. Similarly, harmine and harmaline showed an important inhibition on AChE and weaker BChE inhibitor activity. On the other hand, harmalol and harmol showed more potent BChE inhibitor activity but mediocre AChE inhibitor activity. Also, both nigellastrine I and nigellastrine II gave more solid BChE inhibitor activity but poorer AChE inhibitor activity results. Unlike all, vasicine was influential in inhibiting both AChE and BChE [92]. A new type of alkaloid (2-aldehyde-tetrahydroharmine) was obtained by the use of an activity-guided fraction. It exhibited an AChE and BChE inhibition effect (IC_50_ values with 12.35 ± 0.24 and 5.51 ± 0.33 *μ*M, alternately). On the other hand, vasicine and deoxyvasicine indicated the most powerful BChE inhibition (IC_50_ values of 0.04 ± 0.01 and 0.1 ± 0.01 *μ*M) [55]. A study that used the piezoelectric spraying method showed that the extract of *P. harmala* seed had an AChE inhibitory effect. The study also indicated that the AChE inhibitory effect was two times more potent than the BChE inhibitory effect [93]. Another study used rat liver microsome incubation (RLMs) to show metabolic profiling of deoxyvasicine. Several metabolites were detected. In total, 6 major metabolites were identified. M1, M2-1, and M2-2 were produced for phase I metabolism, and M10-2, M10-3, and M10-4 were produced for phase II metabolism. Also, the inhibitory effect of deoxyvasicine generally varies as per the dosages [94].

#### 5.1.3. Antiviral Activity

The use of nucleoside analogues such as acyclovir is common for the treatment of herpes simplex virus (HSV) infection (genital herpes transmitted by sexual contact) caused by HSV-2. Nevertheless, overuse leads to the growth of resistant strains. None of the extracts from hexane, dichloromethane, and ethyl acetate (except for the methanol extract) of *P. harmala* seeds reached complete inhibition of virus replication with direct contact during and after virus penetration, but there was no activity when this extract was used for the pretreatment of cells. Additionally, the methanol extract on Vero cells did not show a noteworthy cytotoxicity. The combination of harmine and acyclovir compounds were marked to have a synergetic effect against HSV-2 replication [43]. Another study indicated that the *P. harmala* seed extract is also effective against influenza A virus considering the concentration and death rates of Madin-Darby canine kidney (MDCK) cells. The IC_50_ value of the *P. harmala* seed extract on influenza A (H1N1) was 15.7 *μ*g/mL, which is clearly under the recommended IC_50_ value (less than 100 *μ*g/mL) of herbal extracts against infectious diseases [95].

#### 5.1.4. Antioxidant and Antimicrobial Agent

To evaluate the antioxidant value of purified protein (132 kDa) from *P. harmala* seeds, several concentrations of the protein were added to the methanolic DPPH (2,2-diphenyl-1-picrylhydrazyl) solution. The result was that the antioxidant activity of the protein was similar to the antioxidant activity of vitamin C reported [96]. The seed extract of *P. harmala* showed strong monoamine oxidase (MAO-A) inhibitory activity with an IC_50_ value of 27 *μ*g/L based upon harmaline and harmine, while the root extracts' IC_50_ value was 159 *μ*g/L based on just harmine. On the other hand, they exhibited weak MAO-B inhibitory activity [97]. To evaluate the MAO-A and B inhibitors in human liver, ultraperformance liquid chromatography-electrospray tandem mass spectrometry (UPLC-ESI-MS/MS) was used. According to the findings, harmaline, harmine, harmalol, harmane, and tetrahydroharmine indicated important MAO-A inhibitory effects. In contrast, they did not show an MAO-B inhibitory effect [98]. Outcomes from another study, which used a cocktail experiment on human liver enzymes showed that IC_50_ values of harmaline and harmine on MAO-A were 0.1 ± 0.08 *μ*M and 0.38 ± 0.21 *μ*M, respectively. These results showed that harmaline and harmine selectively performed on MAO. In addition, harmaline and harmine were shown to have more potent MAO-A inhibitory activity than AChE [99].

Urinary tract infectious pathogens (*Proteus mirabilis*, *Escherichia coli*, *Pseudomonas aeruginosa*, and *Staphylococcus aureus*) were isolated and studied to determine the antibacterial effect of the seed and leaf extracts of *P. harmala* on them. Antibacterial activity was detected by agar diffusion. Methanolic extracts of *P. harmala* leaves were more robust in inhibiting the microorganisms than the aqueous extracts. On the other hand, *P. harmala* seed extracts were not inhibitory against the growth of *P. mirabilis* and barely inhibitive against the growth of other microorganisms. The applications of the flavonoid extracts of *P. harmala* to *P. aeruginosa* and *S. aureus* were an exception in that regard. Also, this study exhibited that the Gram-positive strain of *S. aureus* was less resilient to flavonoid extracts of seeds and leaves. However, *E. coli* was found to be resistant to the seed and sensitive to the leaf extracts [100].

Another study on the antibacterial and antifungal activities of beta-carboline from *P. harmala* seeds indicated that alkaloids have positive inhibitory interaction when they are applied as binary mixtures. For example, harmine showed the strongest inhibition zone against *Proteus vulgaris* (24.7 mm), *Bacillus subtilus* (21.2 mm), and *Candida albicans* (22.2 mm), while *E. coli* and *Aspergillus niger* were found to be most sensitive to harman with inhibition zones of up to 20.8 mm. Additionally, stronger inhibition zones against *P. vulgaris* (22.6 mm) and *C. albicans* (21.3 mm) were detected when harmaline was applied. However, the application of harmoline presented mediocre activities. On the other hand, when harman and harmaline were used as a binary mixture, *P. vulgaricus* and *C. albicans* inhibition zones reached to 28.9 and 29.0 mm, respectively. A study also showed that the most effective mixture was the whole *β*-carboline fraction with inhibition zones varying from 23.7 to 31.5 mm. The results of this study reported that *E. coli*, *P. vulgaris*, *C. albicans*, and *A. niger* were more sensitive against applied alkaloids, while *S. aureus* was less sensitive. The lowest value for minimal inhibition concentration (MIC) was performed by total (crude) harmala alkaloids (0.333 mg/mL) and binary mixtures of harman with either harmine or harmaline [101]. By the use of the piezoelectric spraying procedure, it was presented in a study that the extract of *P. harmala* seeds had the most antibacterial effect on *Aliivibrio fischeri* [93].

### 5.2. *In Vivo* Studies

#### 5.2.1. Treatment of Respiratory Disorders

The traditional application of *P. harmala* was for treating some respiratory diseases such as cough and asthma. Use of aerial parts of *P. harmala* (APP) in a study showed that when mice with ammonia-induced cough were treated with the total extract of APP (EXT), cough frequency decreased in 2 minutes at all concentrations of 183.3, 550, and 1650 mg/kg, respectively. Also, the alkaloid fraction of EXT (ALK) showed the same inhibitive effect on the cough model at all concentrations of 10, 30, and 90 mg/kg, respectively [63]. However, the flavonoid fraction of EXT did not present a significant reduction in cough frequency [102]. According to the findings of this cough model, EXT and ALK have stronger inhibitory effect on mice at medium and high concentrations than codeine phosphate which was used on the control mice at 30 mg/kg. In addition, EXT and ALK prolonged the latent period of cough at each concentration and were more efficient at medium and high concentrations than codeine phosphate. When ammonia was replaced with capsaicin, EXT and ALK had an obviously stronger effect compared to codeine phosphate on cough frequency at high concentrations. No significant inhibition was detected with the flavonoid fraction (FLA). Moreover, EXT and ALK were a more potent inhibitor for reducing cough frequency in 5 minutes at high concentrations than codeine phosphate when they were applied on mice with citric acid-induced cough. In the meantime, they also prolonged the latent period of cough. High dosages of EXT and ALK from APP were better as an expectorant than ammonium chloride. All of EXT, ALK, and FLA had bronchodilating effects on guinea pigs by prolonging the preconvulsive time [103]. It was indicated by another study that vasicine, vasicinone, and deoxyvasicine from AAP were inhibitive against cough frequency and prolonged the latent period of cough on mice and guinea pigs at a high dosage of 45 mg/kg, which was similar to 30 mg/kg of codeine phosphate. Vasicine, vasicinone, and deoxyvasicine were proven to be strong expectorants on mice. Moreover, vasicinone was more successful at 15 and 45 mg/kg than ammonium chloride. All alkaloids showed obvious bronchodilation effects on guinea pigs depending on their dosages [104].

#### 5.2.2. As Antiulcer Agent

Peganine hydrochloride from *P. harmala* seeds were applied against cold-resistant, aspirin-, alcohol- [6], and pyloric ligation-induced gastric ulcer models in rats to find out its antiulcer activities. According to this research, the antiulcer activity of peganine hydrochloride was significant in rats. Also, peganine hydrochloride was found to be protective in alcohol, potentially cytoprotective in aspirin, and palliative for free and total acidity in pyloric ligation [105].

#### 5.2.3. Improving Brain Function

Two different dosages of harmine were tested on rats with a delayed-match-sample test and water maze task. Harmine showed noteworthy improvement on short-term working and recent memory. On the other hand, dosages of 1 and 5 mg harmine exhibited no influence on spatial reference memory and the administration of high dosage caused motor deficits on many of these rats [106]. Another study showed that harmine was tested on scopolamine- (1 mg/kg) induced Alzheimer's disease mice and in APP/PS1 transgenic mice with Morris Water Maze Test. Application of harmine (20 mg/kg) for 2 weeks substantially enhanced the spatial learning of mice (scopolamine induced). In 10 weeks, harmine exhibited little benefit on APP/PS1 mice. Also, this study demonstrated that harmine could penetrate through the blood-brain barrier and activate Egr-1, c-Fos, and c-Jun [107]. According to real time PCR, Western blot analysis on RBE4 cells (*in vitro*), and alternation of cholinergic neurotransmitters, harmaline and harmine selectively performed on AChE [99].

To evaluate the antidepressive-like effect of acute harmine application, rats were acutely administered with harmine at 5, 10, and 15 mg/kg and imipramine at 10, 20, and 30 mg/kg, respectively, and rats' performances were tested in forced swimming and open-field tests. Treatment of harmine with 10 and 15 mg/kg and imipramine with 20 and 30 mg/kg showed reduction in the period of immobility, while climbing and swimming time increased and did not disturb the locomotor activity. In addition, brain-derived neurotrophic factor (BDNF) protein levels were enhanced in rat hippocampus by acute treatment of harmine at greater dosages [108]. To determine the behavioral and physiological influences of harmine in the chronic mild stress (CMS) rat model, the CMS method was carried out. After 40 days of the CMS method, harmine (15 mg/kg/day) was given to rats for a week. Consequently, anhedonic behavior and hypertrophy of adrenal gland were reversed after harmine treatment. Also, harmine regularized adrenocorticotropic hormone circulating levels and BDNF protein levels [109]. Another study showed that harmine and imipramine had similar behavioral outcomes, while on the contrary, they had dissimilar molecular outcomes [110].

Extract and alkaloid fractions isolated from APP were examined for their antiamnesic effects on mice. Extract and alkaloid fractions proved to have positive effects on learning and memory processes of mice for scopolamine-induced cases. Furthermore, alkaloid fractions were confirmed as major effective components of APP [111].

#### 5.2.4. Antiviral Effect

Another research presented that the extract of *P. harmala* seeds (PHS) were therapeutic against PR8 (H1N1) influenza virus. The study indicated that the PHS extract had an antiviral effect similar to oseltamivir (20 mg/kg). Oral application of 200 mg/kg PHS reduced the loss of body weight and enhanced the rate of survival [95].

**Figure 4:**
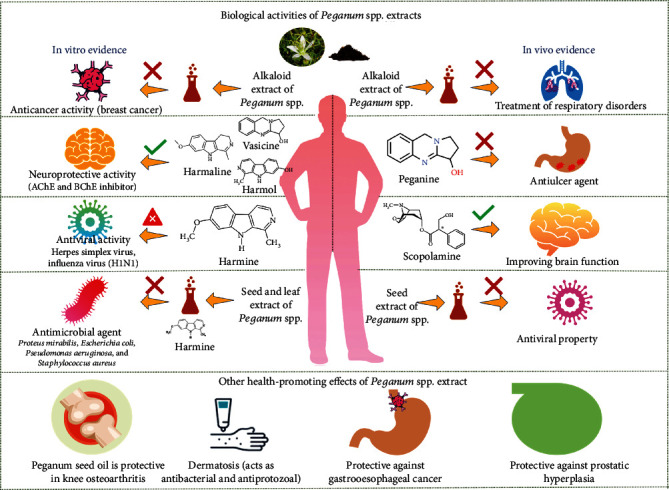
Biological activities and health-promoting effects of *Peganum* spp. extracts.

A comprehensive illustration presenting various bioactivities and health-promoting effects can be seen in Figure 4.

## 6. Health-Promoting Effects Evidenced by Clinical Trials

Despite various experimental studies in both cell-based and animal models, the amount of published clinical trials regarding *Peganum* spp. is scarce. The main findings are mentioned in the following subsections.

### 6.1. Knee Osteoarthritis

A randomized, double-blind, placebo-controlled trial (RCT) evaluated the efficacy of a standardized preparation of *P. harmala* oil in pain management, stiffness, tenderness, and function after four weeks of treatment [102]. There was a significant pain reduction in the intervention group (52.56% vs. 17%, *P* < 0.05) according to the Visual Analogue Scale (VAS) and Western Ontario and McMaster Universities Arthritis Index (37.89% vs. 16.41%, *P* < 0.001). Regarding function, there was an 80% decrease in pain on motion factor in patients using the oil, which was significant when compared to the placebo (22.2%, *P* < 0.01). Similarly, tenderness improved by 65% compared to the control group (14.8%, *P* < 0.001). There was no significant improvement in stiffness. These results, while promising, require further examination due to their limited external validity, considering the small sample size (*n* = 54), and only 3 of the patients recruited were males.

### 6.2. Dermatoses

A clinical trial performed in Egypt evaluated the antibacterial and antiprotozoal activity of *P. harmala* in multiple skin conditions [112]. One hundred and eleven patients with multiple or bilateral lesions were randomly selected and followed for four weeks. All patients received the intervention, an ointment with alkaloids extracted from the plant in question and a placebo. They used the ointment with the active ingredient on one side while they used the placebo on the other. All patients with impetigo (*n* = 20) showed significant improvements in symptoms and laboratory results. Of all patients with pityriasis alba (*n* = 18), 72% had clinically significant improvement. Fifty percent of patients with tinea circinata (*n* = 20) improved considerably, albeit fungi were detected in cultures of 10% of them. Concerning leishmaniasis, the response was favorable in 5 of the seven patients with this disease; however, the lesions were not cleared completely. Patients with itching dermatoses (i.e., Lichen planus, Lichen simplex chronicus) showed a notable change in their symptoms. Psoriasis did not improve at all; in fact, treatment was discontinued during the third week. Placebo did not produce any improvement. These results show a promising use of *P. harmala* as an antibacterial, antipruritic, and antifungal agent. Nevertheless, it is necessary to evaluate the efficacy of the intervention on a grander scale, explicitly focused on each of the dermatoses with a clear description of the inclusion and exclusion criteria.

### 6.3. Gastroesophageal Cancer

Three studies evaluated the therapeutic and adverse events of Spinal-Z, with *P. harmala* and *Dracocephalum kotschyi* Boiss as its main components, in patients with gastroesophageal cancer. It is used as palliative therapy in combination with standard treatment. The initial study evaluated the response to Spinal-Z in seven patients with cancer in the upper gastrointestinal tract, during a nine-month follow-up [113]. Of these seven patients, one showed a reduction in the growth of metastases. Common side effects included dizziness and vomiting. One patient died of drug-induced hepatitis. Although causality with Spinal-Z was not proven, it should be evaluated in further studies. The remaining studies [114, 115] presented larger sample sizes; one was focused on gastroesophageal cancers in general (*n* = 61), while the other was focused on patients with metastatic gastroesophageal adenocarcinoma (*n* = 76). Both studies showed meaningful improvement in gastrointestinal symptoms such as dysphagia, heartburn, poor appetite, nausea, and constipation (*P* < 0.05). There were no significant changes in laboratory findings (*P* > 0.05) or drug-related adverse symptoms, except for muscle weakness in one of the studies (*P* < 0.05).

### 6.4. Benign Prostatic Hyperplasia

In a clinical trial study, 90 patients with benign prostatic hyperplasia with lower urinary tract symptoms were randomized into three groups: patients receiving an oral capsule of *P. harmala*, a second group receiving tamsulosin, and a third group receiving combined therapy (*P. harmala* plus tamsulosin) [116]. Efficacy was evaluated based on the International Prostate Standard Survey (IPSS) that evaluates seven symptoms: incomplete discharge, urinary frequency, intermittency, urgent need to urinate, weak flow of urine, straining to urinate, and nocturia. All groups had a statistically significant change in mean IPSS scores after four weeks of treatment (*P* < 0.001). However, there was no significant difference between groups after treatment, with the exception of urinary frequency, intermittency, and nocturia. The combined therapy group seemed to have more effect on alleviating symptoms, although the study does not report whether these results are significant or not. Also, there is no report on adverse events on neither of the groups. Finally, blinding methods are not clearly explained. In the summary section, the article indicates that it is a single-blind study; yet it indicates that it is a double-blinded study on the methods sections. Either way, there is no clear indication of how blinding was performed.

## 7. Safety and Adverse Effects

As mentioned throughout this manuscript, for *P. harmala*, the whole plant and various parts of this plant, including its seeds, bark, and roots, have been used as traditional medicine in Iran along with many other countries. Recent researchers have shown different pharmacological and therapeutic effects of *P. harmala* and its active alkaloids, especially harmine and harmaline [29].

This plant has also been studied in relation to its bactericidal activity, as a natural alternative where the total alkaloid extract of *P. harmala* seeds was tested *in vitro* on four phytopathogenic bacteria, namely, *Ralstonia solanacearum* Phylotype II, *Erwinia amylovora*, *Pectobacterium carotovorum* subsp. *carotovorum*, and *Burkholderia gladioli*, the causal agents of potato brown rot, pear fire blight, potato soft rot, and onion slippery skin diseases, respectively [117]. Hence, the MIC and the minimum bactericidal concentration (MBC) were evaluated *in vitro*, obtaining the MBC of 150 *μ*g/mL for *R. solanacearum*, followed by *B. gladioli* (MBC 200 *μ*g/mL). The extract exhibited a marked inhibitory effect *in vitro* on the pathogen *R. solanacearum* at concentrations ranging from 4 to 300 *μ*g/mL, but this effect on the other pathogens required higher concentrations (50–300 *μ*g/mL). However, it was observed that *R. solanacearum* cells exposed to 4 *μ*g/mL presented severe cell damage and genome coagulation, as well as a disorganized cytoplasm and a thickened cell wall compared to the control. In general, this study revealed the antibacterial efficacy of the total alkaloid extract of *P. harmala* on phytopathogenic bacteria that could be used as an alternative for chemical antibacterials [117]. Nevertheless, in order to revise the cytotoxic response in bacteria, it would be necessary to deepen the adverse effects in humans.

On the other hand, in a study guided in this sense, the Algerian seeds of *P. harmala* were evaluated for use in therapeutic and clinical trials as mentioned above. In parallel with these trials, there have been some studies on adverse effects and safety that are important to review in this section. Among them, an oral administration of *P. harmala* extract was carried out in mice at a dose of 0–12 g/kg to evaluate the acute toxicity causing adverse effects at an LD_50_ of 2.86 g/kg [118]. In a study to evaluate the subacute effects carried out by the daily oral administration of an aqueous extract for 28 consecutive days, no mortality was obtained, although a significant difference was observed in the weight of the organs between the control animals and those that received the treatment. At a biochemical level, significant differences were also observed in bilirubin, uric acid, and alkaline phosphatase as well as in the hematological analysis, where differences were also observed in the leukocyte count and the estimation of hemoglobin. Only the urinalysis was negative for all parameters except glucose. In addition, from the pathological point of view, some serious abnormalities and histological changes were observed in liver and kidney tissues [118]. These results give us an idea of the high activity of the alkaloids contained in this plant and therefore of the need to deepen the knowledge of the adverse effects that they could present in humans.

## 8. Conclusions

Future perspectives of new bioactive compounds and development of new drugs have been focusing on natural products. As it has been presented throughout the manuscript, harmine and other alkaloids found in the genus *Peganum* could be used for pharmaceutical and clinical purposes since they provide health benefits and significant effects on treatments of diseases.

These plants have the advantage of functioning as bactericides and virucides and have therapeutic effects showing wide health benefits on humans. Nevertheless, despite all the therapeutic potential, at higher doses and long periods of exposure, extracts from *Peganum* can cause several cytotoxic effects such as several hepatic and nephritic toxicities. In this respect, all new drugs need to be studied in depth, even natural ones, since the adverse effects of these plants also need to be described. Therefore, nanotechnology, development of new drugs, and designing new formulations where release of active compounds may be controlled are necessary to achieve successfully pharmacological and therapeutically treatments.

## Figures and Tables

**Table 1:** Distinctive morphological characteristics of *Peganum* species.

Morphological characteristics	*P. harmala*	*P. multisectum*	*P. nigellestrum*	References
Plant height	40-45 cm	20-65 cm	10-25 cm	[13, 24, 27, 28]
Stem and leaf surface	Glabrous	Sparse setose	Dense setose	
Stem branching	Branched into 5-13 stems	Branched into 70-100 stem	Branched or without branched	
Leaves	Palmatisected into 3-5 linear lobes; lobes 1.5-3.0 mm wide	Bi- or tripalmatisected into linear lobes; lobes 0.4-1.0 mm in width	Bi- or tripalmatisected into linear lobes; lobes 1.2-1.8 mm in width	
Calyx leaves	Calyx leaves entire or slightly incised	Calyx leaves incised into 3 linear lobes	Calyx leaves incised into 5-7 linear lobes	
Seed	Depressed surface	Convex surface	Smooth surface	

**Table 2:** Traditional uses of *Peganum* species.

*Peganum* spp.	Part of plant	Country/region	Common name	Effect	Reference
*P. harmala*	Seeds	Morocco	Madjouna	Diabetes, asthma, rheumatic pain, antihypertensive, anthelmintic/antimicrobial	[29, 31, 32]
*P. harmala*	Seeds, fruit	Iran/Uzbekistan	Asbatan, fašars ī n, isfanj, ispand, sifand, sipand, isirik	Air purifier, pain relief, anti-Parkinson's	[3, 29, 32]
*P. harmala*	Seeds, fruit	Turkey	Ilezik, uzarih, üzerlik	To treat intestinal pain, as an antibacterial, necklaces (sometimes also a branch of the plant is hung in the house)	[29, 33]
*P. harmala*	Seeds	Arabian (Jordan, Saudi Arabia, Syria)	Álqat al-dib, harjal, harmal, hre-milan, huraymilan, legherma, mogannanna, sadab-sami, sadab-bari	Common cold, as a dye, healing ulcers, back pain, blood purifier, psychoactive	[29, 32]
*P. harmala*	Whole plant, seeds,	Greece, Spain, Italy	Alfarma, armalá, gamarza, harma, hármaga, harmala, African rue, harmal, harmal peganum, harmal shrub, harmel, harmel peganum, Isband, ozallaik, peganum, rue, ruin weed, Syrian rue, wild rue, alfarma, alharma, amargaza, armalá, gamarza, harma, hármaga	Hypotensive, anti-Parkinson's, antidiabetic, leishmaniasis	[29, 34, 35]
*P. harmala* *P. nigellastrum* *P. multisectum*	Seeds	China	Luo tuo peng, luo tuo hao	Hypertension, diabetes, jaundice	[3, 29, 32]
*P. harmala*	Seeds	India	Hurmul	Asthma	[30, 32]
*P. harmala* *P. multisectum*	Seeds	Mexico, southern North America	Isband, ozallaik, peganum, rue, ruin weed, Syrian rue, wild rue	Nervosity	[29, 36]
*P. harmala*	Seeds	North of Africa	African rue, huraymilan, legherma, mogannanna, sadab-sami, sadab-bari	Emmenagogue	[29, 37]

**Table 3:** Essential oil composition (%) of *Peganum harmala* L. from different regions.

Compound name	Algeria	Egypt	Libya	Morocco	Tunisia	Iran	S. Arabia	Morocco
	[37]	[37]	[37]	[37]	[37]	[40]	[41]	[39]
*p*-Cymene	—	—	—	—	0.1	0.8-1.7	—	—
Limonene	0.3	—	—	—	—	1.1-6.4	—	—
2-Acetyl-thiazole	—	1.3	—	—	—	—	—	—
Santolina alcohol	—	2	—	—	—	—	—	—
*cis*-Dihydro-rose oxide	—	1.8	—	—	—	—	—	—
1-Octen-ol	—	0.7	—	—	—	—	—	—
*trans*-Dihydro-rose oxide	—	0.4	—	—	—	—	—	—
*n*-Octanol	—	1.1	—	—	—	—	—	—
Linalool	1.7	—	—	—	—	0.4	—	—
*trans*-Thujone	—	—	—	—	0.1	—	—	—
Methyl butanoate, 3-methyl-3-butenyl	—	2.9	—	—	—	—	—	—
3-Decanone	—	1.1	—	—	—	—	—	—
Camphor	—	2.7	—	—	—	—	—	—
Benzene acetonitrile	—	1.3	—	—	2.6	—	—	—
Isoborneol	0.9	—	—	—	—	—	—	—
Terpinen-4-ol	0.4	—	—	—	—	—	—	—
Naphthalene	0.2	—	—	—	—	—	—	—
*α*-Terpineol	0.7	—	—	—	—	—	—	—
1-Dodecene	—	0.7	—	—	—	—	—	—
[2]-Isocitral	0.7	—	—	—	—	—	—	—
Methyl chavicol	1.1	—	—	—	0.1	—	—	—
Isoquinoline	0.5	—	—	—	—	—	—	1.4-6.4
Carvone	0.7	—	—	—	—	—	—	—
Pulegone	0.5	—	—	—	—	—	—	—
Cumin aldehyde	—	—	—	—	0.1	—	—	—
[2]-Anethol	3.7	—	—	—	6.9	—	—	—
*α*-Terpinen-7-al	—	2.2	—	1	0.1	—	—	—
Thymol	7	—	5.1	5	1.8	—	—	2
Dihydrocarveol acetate	6.2	1	3	0.7	0.3	—	—	—
Terpinyl acetate	—	0.9	—	—	—	—	—	—
4-Methoxyacetophenone	1	—	—	—	—	—	—	—
Eugenol	17.5	17.2	17.8	13.2	69.2	—	5.2	—
Cicloysosativene	2.3	—	—	—	—	—	—	—
*α*-Ylangene	1.1	—	—	—	—	—	—	—
Decanoic acid	—	1	—	—	—	—	—	—
Thujico acid	—	—	—	—	<0.1	—	—	—
*β*-Cubebene	—	—	—	0.4	—	—	—	—
*n*-Undecanol	—	2.3	—	—	—	—	—	—
6,8-Nonadien-2-one,6-methyl-5-(1-methyletildene)	0.3	—	—	—	—	—	—	—
*iso*-Italicene	1.1	—	—	—	—	—	—	—
*β*-Longipinene	0.5	—	—	—	0.1	—	—	—
Methyleugenol	—	0.3	—	—	—	—	—	—
*β*-Ionol	—	—	—	0.7		—	—	—
(Z)-Caryophyllene	2.3	—	0.3	—	0.8	—	—	—
[2]-*α*-Ionone	0.6	—	—	0.2	—	—	—	—
Nerol acetate	—	—	3.7	—	—	—	—	—
*α*-Isomethyl-[2]-ionol	7	—	—	—	—	—	—	—
Aromadendrene	—	—	—	—	0.3	—	—	—
Bakerol	—	—	—	7.5	—	—	—	—
*trans*-Cadina-16,4-diene	—	—	—	0.3	—	—	—	—
9-*epi*-[2]-Caryophyllene	—	—	—	—	0.2	—	—	—
*α*-Acoradiene	0.5	—	—	—	—	—	—	—
*γ*-Gurjunene	—	—	—	0.6	—	—	—	—
*γ*-Muurolene	0.3	—	—	—	—	—	—	—
[2]-*β*-Ionone	—	—	—	0.6	—	—	—	8.1
[2]-Methyl isoeugenol	—	0.6	—	—	0.2	—	—	—
10,11-Epoxy-calamenene	—	—	—	0.3	—	—	—	—
*α*-Zingiberene	0.3	—	—	—	—	—	—	—
*γ*-Amorphene	1.3	—	—	—	—	—	—	—
Methyl *p*-*tert*-buthylphenil acetate	0.3	0.8	—	2	0.2	—	—	—
10-Undecenol acetate	2	—	—	3.6	0.1	—	—	—
*β*-Curcumene	—	1.9	—	—	—	—	—	—
7-epi-*α*-Selinene	1.1	—	—	—	—	—	—	—
2E,4E-Dodecandienal	1.2	2.1	2	—	—	—	—	—
*α*-Thujaplicinol	—	—	—	4.8	—	—	—	—
Eugenol acetate	—	—	—	—	9	—	—	—
*α*-Bulnesene	—	—	—	0.4	—	—	—	—
(Z)-Nerolidol	0.5	1.6	2.5	1.3	0.1	—	—	—
*α*-Calacorene	0.6	—	—	0.6	—	—	—	—
Germacrene B	—	—	2.7	—	0.1	—	—	—
*β*-Calacorene	—	—	—	1.8	—	—	—	—
Dodecanoic acid	—	5.9	—	—	—	—	—	—
Spathulenol	2	2.3	4.2	4	0.2	—	—	—
Caryophyllene oxide	1.7	—	3.8	1.7	0.8	—	—	—
1-Esadecene	—	1.6	—	0.9	0.2	—	—	—
Ledol	—	—	—	0.4	—	—	—	—
*β*-Oplopenone	—	—	—	—	0.1	—	—	—
*n*-Exadecane	2.6	—	2.8	2.9	0.1	—	—	—
Isolongifolan-7-*α*-ol	—	—	3.8	2.1	0.1	—	—	—
Cubenol	—	2	—	—	<0.1	—	—	—
Caryophylla-4(12),8(13)-dien-5*α*-ol	—	—	—	—	0.1	—	—	—
epi-*α*-Cadinol	5.3	—	—	0.9	—	—	—	—
*β*-Acorenol	1.5	2.9	7.4	2.3	0.1	—	—	—
*epi*-*α*-Muurolol	—	—	—	—	0.1	—	—	—
*cis*-Guai-3,9-dien-11-ol	0.7	—	—	—	—	—	—	—
*allo*-Aromadendrene epoxide	—	—	—	1.5	—	—	—	—
Vulgarone B	1.4	—	—	3.7	—	—	—	—
Cedr-8,15-en-10-olo	—	4.1	6	—	0.1	—	—	—
*α*-Eudesmol	—	1.6	—	—	—	—	—	—
14-Hydroxy-(Z)-caryophyellene	—	—	2.1	—	0.2	—	—	—
*n*-Tetradecanol	4.8	12.3	11.3	11.1	0.3	—	—	—
Elemol acetate	0.7	—	—	—	—	—	—	—
*epi*-*α*-Bisabololo	—	3	6.6	—	—	—	—	—
Cyperotundone	1.6	3	4.6	4.1	0.1	—	—	—
Heptadecane	—	1.2	3.8	1.2	0.1	—	—	—
Calamenen-10-one	—	0.5	1.9	—	—	—	—	—
Longifolol	—	2.7	—	—	—	—	—	—
Farnesol	—	—	—	0.4	—	—	—	—
3-Otadecine	0.3	—	—	—	—	—	—	—
Farnesale	—	1	—	—	—	—	—	—
Santalol	—	—	—	1	—	—	—	—
[2]-Nerolidil acetate	—	—	—	0.8	—	—	—	—
Amorpha-4,9-diene	—	0.3	—	1.6	—	—	—	—
Lanceol	—	—	—	1.5	—	—	—	—
14-Oxy-*α*-muurolene	—	—	—	0.3	—	—	—	—
*α*-Pinene	—	—	—	—	—	60.4-72.6	—	—
Camphene	—	—	—	—	—	1.8-1.1	—	—
Verbenene	—	—	—	—	—	0.9	—	—
Sabinene	—	—	—	—	—	2.6	—	—
*β*-Pinene	—	—	—	—	—	0.7-2.5	—	—
*α*-Campholene-aldehyde	—	—	—	—	—	1.7	—	—
*trans*-Pinocarveol	—	—	—	—	—	2.3	—	—
*trans*-Verbenole	—	—	—	—	—	3.9	—	—
*cis-*Verbenole	—	—	—	—	—	1.2	—	—
Karahanaenone	—	—	—	—	—	0.7	—	—
Pinocamphone	—	—	—	—	—	0.5	—	—
Pinocarvone	—	—	—	—	—	0.7	—	—
*p*-Mentha-1,5-dien 8-ol	—	—	—	—	—	1.7	—	—
Terpineol-4	—	—	—	—	—	0.5	—	—
Myrtenal	—	—	—	—	—	1	—	—
Verbenone	—	—	—	—	—	1.8	—	—
Bornyl acetate	—	—	—	—	—	0.5	—	—
Monoterpene hydrocarbons	—	—	—	—	—	72.9-81.6	—	—
Oxygen-containing monoterpenes	—	—	—	—	—	14.5	—	—
Oxygen-containing sesquiterpenes	—	—	—	—	—	2.4	—	—
2-Octene	—	—	—	—	—	2.3	—	—
3-Octaodiene	—	—	—	—	—	0.7	—	—
2-Methyl-phenol	—	—	—	—	—	0.8	—	—
Methyl benzene	—	—	—	—	—	2.1	—	—
Dimethylbenzene	—	—	—	—	—	2	—	—
Styrene	—	—	—	—	—	4.2	—	—
Nonane	—	—	—	—	—	1.7	—	—
Propyl benzene	—	—	—	—	—	1.9	—	—
1-Hexyl-2-nitrocyclohexane	—	—	—	—	—	—	9	—
(Z)-2-Octadecen-1-ol	—	—	—	—	—	—	8.1	—
3,5,24-Trimethyltetracontane	—	—	—	—	—	—	7.8	—
2-Octadecyl-1,3-propane-diol	—	—	—	—	—	—	6.1	—
[2]-2-Tetradecen-1-ol	—	—	—	—	—	—	5.8	—
11,14-Eicosadienoic acid methyl ester	—	—	—	—	—	—	5.7	—
2,6,10,15-Tetramethyl-heptadecane	—	—	—	—	—	—	4.2	—
11-Tricosene	—	—	—	—	—	—	4	—
2-Piperidinone, n-[4-bromo-n-butyl]	—	—	—	—	—	—	3.4	—
14-Heptadecenal	—	—	—	—	—	—	2.8	—
[2]-9-Tetradecenoic acid	—	—	—	—	—	—	2.7	—
Propylic acid	—	—	—	—	—	—	—	2.8-46.3
Butanol	—	—	—	—	—	—	—	3.5
Pent-3-en-2-one	—	—	—	—	—	—	—	3.2-4.9
Butyric acid	—	—	—	—	—	—	—	5.0-7.5
[2]-3-hexenol	—	—	—	—	—	—	—	2.2
Methy-4-methyl valerate	—	—	—	—	—	—	—	0.9-2.3
Tiglic acid	—	—	—	—	—	—	—	13.6

**Table 4:** Phytochemicals present in different parts (seeds, roots, stems, fruits, flowers, leaves, testa, and whole plant) of *Peganum harmala* L. and overall profile of *Peganum multisectum* (Maxim.) Bobrov and *Peganum nigellastrum* Bunge.

Plant parts	Phytoconstituents	References
*Peganum harmala* L.		

Seeds	Hexadecanoic acid; methyl linoleate; methyl oleate; 9-octadecenoic acid; ethyl linoleate; ethyl oleate; harmaline; harmine; delta-tocopherol	[42]
	Harmine	[43]
	Harmaline; harmine; asparagine; sucrose; choline; phosphorylcholine	[44]
	Harmine; vasicinone; pegamine; H3-hydroxylated; pegamine dimer; ruine; deoxyvasicinone; tetrahydroharmin; peganine; harmaline; harmalol; pegaline; dexoypeganine	[45]
	2-Oxoindole alkaloids; (±)-peganumalines A-E; peganumaline	[46]
	*p*-Coumaric acid; rutin; catechin; hesperetin; chloregenic acid	[47]
	Peganine A; peganine B; peganumal A; peganumal B; pegaharmine F; pegaharmine G; pegaharmine H; pegaharmine I; pegaharmine J; pegaharmine K	[48]
	Peganine B; peganumal A; peganumal B; pegaharmine F; pegaharmine G; pegaharmine H; pegaharmine I	[49]
	3*α*-Acetoxy-27-hydroxyolean-12-en-28-oic acid methyl ester; syringin; [5]-caffeyl alcohol 4-O-*β*-D-glucopyranoside; coniferin; syringinoside; N-acetyl-9-syringinoside	[50]
	Pegaharmine A; pegaharmine B; pegaharmine C; pegaharmine D; pegaharmine E	[51]
	Peganone I (3,6-dihydroxy-8-methoxy-2-methylanthraquinone); peganone II (8-hydroxy-7-methoxy-2-methylanthraquinone)	[52]
	Harmine; harmaline; harmol; harmalol; harmane; norharmane	[53]
	Indole; quinoline,2,3,4-trimethyl-; tetrahydroharman; oleanitrile; tetrahydroharmine; harmaline; harmine; 9-octadecenamide, (z)-; 6-methoxytetrahydro-1-norharmanone; 4-amino-2-ethyl-3 methylquinoline	[54]
	2-Carboxyl-3,4-dihydroquinazoline; 3-hydroxylated harmine; 1-hydroxy-7-methoxy-*β*-carboline; acetylnorharmine; harmic acid methyl ester; 2-aldehyde-tetrahydroharmine; harmalanine; harmine *N*-oxide; 2-carboxyl-3,4-dihydroquinazoline; 6-methoxyindoline; 1-O-*β*-D-xylopyranose sinapyl alcohol	[55]
	[5]-Vasicinone-Glu; [5]-vasicinone-Glu	[56]
	3-Hydroxy-3-(N-acetyl-2-aminoethyl)-6-methoxyindol-2-one; 11-methyoxyl-rutaecarpine	[57]
	Harmic acid; 4,5-dihydroblumenol	[58]
	Oleic acid; linoleic acid ((Z,Z)-9,12-octadecatrienoic acid); linolenic acid; palmitic acid (hexadecanoic acid); arachidic acid	[59]
	Peganumine A	[60]
	Peganumine A; peganumine B	[61]
	Harmalidine	[62]
	Harmalacidine	[63]
	Diglycoside vasicine; vasicine; vasicinone; harmalol; ruine; harmol; 8-hydroxy-harmine; tetrahydroharmine; harmaline; harmine	[64]
	Norharmane	[65]
	Harmane; harmol; harmine	[66]
	Tetrahydroharmine	[67]
	Harmine; harmaline; vasicine; vasicinone	[68]
	Quercetin-3-*O*-gentiobioside; quercetin-3-*O*-rutinoside; kaempferol-3-methyoxyl-5-*O*-rutinoside; kaempferol-3,5-dimethyxyl-7-*O*-glucoside; stearic acid; kaempferol-3-methyoxyl-7-*O*-glucoside; isorhamnetin-7-*O*-glucoside; isorhamnetin-3-*O*-rutinoside; anthraquinone glucoside; 9,14-dihydroxyoctadecanoic acid	[69]
	Dodecane; tetradecane; methyl dodecanoate; hexadecane; 2-octanol benzoate; heptadecane; methyl tetradecanoate; 2,6,10,14-tetramethyl pentadecane; octadecane; 2,6,10,14-tetramethyl hexadecane; nonadecane; methyl hexadecanoate; dibutyl phthalate; eicosane; methyl oleate; henicosane; docosane; harmine; tricosane	[70]
	Acacetin 7-0-rhamnoside; acacetin 7-0-[6^″^-0-glucosyl-2^″^-O-(3^‴^-acetylrhamnosyl)] glucoside; acacetin 7-O-(2^‴^-O-rhamnosyl-2^″^-O-glucosyl)glucoside; 2^‴^-0-rhamnosyl-2^″^-0-gluco-sylcytisoside	[71]
	Ruine; dihydroharmane; dihydroruine; tetrahydroharmol; harmalicine	[72]

Roots	Threonine; acetic acid; lysine; proline; phosphorylcholine; sucrose; *β*-glucose; formic acid; asparagine; 4-hydroxyisoleucine	[44]
	Harmol	[66]
	Luotonin A; luotonin B	[73]
	Luotonin E; luotonin F	[74]
	Vasicol	[75]
	Harmine; vasicine; vasicinone; harmaline	[76]

Flowers	Harmine; peganine	[77]
	Proline; lysine; asparagine; 4-hydroxyisoleucine; sucrose; vasicine; choline; phosphorylcholine	[44]

Whole plant	1-Octadecene; 6,10,14-trimethyl-2-pentadecanone; [5]-15-heptadecenal; oxacyclohexadecan-2-one; 1,2,2,6,8-pentamethyl-7-oxabicyclo[4.3.1]dec-8-en-10-one; hexadecane-1,2-diol; eicosan-3-ol; tetradecanoic acid; 12-methyl tetradecanoic acid; pentadecanoic acid; 5,9,13-trimethyl tetradecanoic acid; tridecanoic acid; 2-methyl-octadecanoic acid; heptadecanoic acid; [5]-9-dodecenoic acid; (Z)-9-hexadecenoic acid; (Z,Z,Z)-9,12,15-octadecatrienoic acid	[78]
	Harmine; harmaline	[77]
	Deoxyvasicine; deoxyvasicinone	[72]

Leaves	Trigonelline; formic acid; vasicinone; vasicine; harmaline; harmine; maleic acid; sucrose; *α*-glucose; *β*-glucose; malic acid; proline; asparagine; betaine; choline; phosphorylcholine; succinic acid; 4-hydroxyisoleucine; acetic acid; alanine; threonine; valine; isoleucine; malic acid; lysine	[79]
	Isoleucine; valine; alanine; betaine; maleic acid; proline; lysine; asparagine; 4-hydroxyisoleucine; acetic acid; sucrose; vasicine; choline; phosphorylcholine	[44]
	Aspartate; threonine; serine; glutamate; glycine; alanine; valine; methionine; isoleucine; leucine; tyrosine; phenylalanine; lysine; histidine; arginine; proline; cystine	[73]

Stem	Lysine; succinic acid; malic acid; vasicinone; proline; asparagine; 4-hydroxyisoleucine; asparagine; sucrose; vasicine; choline; phosphorylcholine	[44]

Areal part	10-Vasicinol; 11-vasicinol; 4-vasicinol; 6-vasicinol; vasicine-glu; vasicine-2glu; vasicine-2glu; methylation-acetylation-vasicine; methylation-acetylation-vasicine; vasicinol-glu; vasicinone-glu; vasicinone-2glu; vasicinone-2glu; pegaline; diosmetin+3glu+rha+ac; acacetin+glu+rha; diosmetin+glu+rha	[75]
	2-Ethoxylpropane; 3-hydroxy-3-methyl-2-butanol; methylcyclopentane; 2-methylhexane; 3-methylhexane; 1-ethoxy-2-methylpropane; heptane; methylcyclohexane; 2,4-dimethylhexane; 1-octen-3-ol; 2-methylheptane; 3-methylheptane; hexanol; *cis*-1,3-dimethylcyclohexane; 2-octanol; octane; *cis*-1,4-dimethylcyclohexane; [5]-2-hexanol; 1,3-dimethylbenzene; benzaldehyde; 6-methyl-5-heptane-2-one; [8]-[5]-5-ethyl-2(5H)-furanone; 3,5,5-trimethyl-2-cyclohexene-1-one; 12-heptadecyn-1-ol; benzenamine; N-phenyl-formamide; geranylacetone; 1,2,3,3,4-pentamethylcyclopenene; 5,6,7,7*α*-tetratydro-4,4,7*α*-trimethyl-2(4H)-benzenofranone; 6,10,14-trimethyl-2-pentadecanone; 3,7,11,15-tetramethyl-2-hexadecen-1-ol; dodecanoic acid; hexadecanoic acid	[80]
	Liriodendrin; *trans*-ferulic acid *β*-D-glucopyranoside; (*6S,7E,9R*)-6,9-dihydroxymegastigma-4,7-dien-3-one-9-*O*-*β*-D-glucopyranoside (or roseoside); (*3S,5R,6R,7E,9S*)-megastigman-7-ene-3,5,6,9-tetrol-3-O-*β*-D-glucopyranoside	[81]
	Deoxypeganidine; peganidine; peganol; quinoline; pegamine; hemicellulose; gentisate-2,5-diglucoside	[72]

Fruits	*α*-Glucose; choline; proline; lysine; asparagine; 4-hydroxyisoleucine; acetic acid; sucrose; vasicine; choline; phosphorylcholine	[44]
	Harmine; peganine; harmaline	[77]

Testa	4-Hydroxyisoleucine; asparagine; proline; vasicine	[44]

Floral nectar	Harmalol; harmine; fructose; glucose; sucrose; aspartic acid; glutamic acid; serine; glutamine; glycine; histidine; alanine; proline; tyrosine; valine; phenylalanine	[82]

*Peganum multisectum* (Maxim.) Bobrov	2-Methylquinoline; 9-amino-2,3,5,6,7,8-hexahydro-1H-cyclopenta [b] quinoline; vasicinone; harmine; peganine; deoxypeganine; deoxyvasicinone; harmane	[83]
	Vasicine	[84]
	(*S/R*)-Vasicinone	[10]
	Harmol; harmane; harmine; harmaline; harmalol	[66]
	Peganetin; deacetylpeganetin; 7,4′-dihydroxy-3′-methoxy-5-O-rutinoside	[85]
	Aspartate; threonine; serine; glutamate; glycine; alanine; valine; methionine; isoleucine; leucine; tyrosine; phenylalanine; lysine; histidine; arginine; proline; cystine	[73]
	2-Ethoxylpropane; 3-hydroxy-3-methyl-2-butanol; methylcyclopentane; 2-methylhexane; 3-methylhexane; 1-ethoxy-2-methylpropane; heptane; methylcyclohexane; 2,4-dimethylhexane; 1-octen-3-ol; 2-methylheptane; 3-methylheptane; hexanol; *cis*-1,3-dimethylcyclohexane; 2-octanol; octane; *cis*-1,4-dimethylcyclohexane; [5]-2-hexanol; 1,3-dimethylbenzene; benzaldehyde; 6-methyl-5-heptane-2-one; [8]-[5]-5-ethyl-2(5H)-furanone; 3,5,5-trimethyl-2-cyclohexene-1-one; 12-heptadecyn-1-ol; benzenamine; N-phenyl-formamide; geranylacetone; 1,2,3,3,4-pentamethylcyclopenene; 5,6,7,7*α*-tetrahydro-4,4,7*α*-trimethyl-2(4H)-benzenofranone; 6,10,14-trimethyl-2-pentadecanone; 3,7,11,15-tetramethyl-2-hexadecen-1-ol	[80]
	Deoxyvasicine; deoxyvasicinone	[72]

*Peganum nigellastrum* Bunge	Vasicine	[64]
	(*S/R*)-Vasicinone	[10]
	Diosmetin 7-O-*β*-D-glucopyranosyl(1→2)-*β*-D-glucopyranosyl(1→2)-[*α*-L-rhamnopyranosyl (1→6)]-*β*-D-glucopyranoside	[86]
	Nigellastrine I; nigellastrine II; harmol; harmane; harmine; harmaline; harmalol	[66]
	Dihydrosinapyl ferulate; dihydroconiferyl ferulate	[36]
	3*α*,27-Dihydroxylup-20(29)-en-28-oic acid methyl ester; 3*α*-acetoxyolean-12-ene-27,28-dioic acid 28-methyl ester; 3-oxotirucalla-7,24-dien-21-oic acid; 3*α*-acetoxy-27-hydroxylup-20(29)-en-28-oic acid methyl ester; betulinic acid; 3-O-acetylbetulinic acid; 3-epibetulinc acid; 3-O-acetylepibetulinic acid	[87]
	Acacetin; peganetin; deacetylpeganetin	[85]
	Aspartate; threonine; serine; glutamate; glycine; alanine; valine; methionine; isoleucine; leucine; tyrosine; phenylalanine; lysine; histidine; arginine; proline; cystine	[73]
	2-Ethoxylpropane; 3-hydroxy-3-methyl-2-butanol; methylcyclopentane; 2-methylhexane; 3-methylhexane; 1-ethoxy-2-methylpropane; heptane; methylcyclohexane; 2,4-dimethylhexane; 1-octen-3-ol; 2-methylheptane; 3-methylheptane; hexanol; *cis*-1,3-dimethylcyclohexane; 2-octanol; octane; *cis*-1,4-dimethylcyclohexane; [5]-2-hexanol; 1,3-dimethylbenzene; benzaldehyde; 6-methyl-5-heptane-2-one; [8]-[5]-5-ethyl-2(5H)-furanone; 3,5,5-trimethyl-2-cyclohexene-1-one; 12-heptadecyn-1-ol; benzenamine; N-phenyl-formamide; geranylacetone; 1,2,3,3,4-pentamethylcyclopenene; 5,6,7,7*α*-tetrahydro-4,4,7*α*-trimethyl-2(4H)-benzenofranone; 6,10,14-trimethyl-2-pentadecanone; 3,7,11,15-tetramethyl-2-hexadecen-1-ol;	[80]
	Deoxyvasicine; deoxyvasicinone	[72]

## References

[1] Shamsa F., Monsef H. R., Ghamooghi R., Verdian R. M. R. (2007). Spectrophotometric determination of total alkaloids in *Peganum harmala* using bromocresol green. *Research Journal of Phytochemistry*.

[2] Goel N., Singh N., Saini R. (2009). Efficient in vitro multiplication of Syrian Rue (*Peganum harmala*) using 6-benzylaminopurine preconditioned seedling explants. *Natural Science*.

[3] Mina C. N., Mohammad H. F., Gholamreza A. (2015). Medicinal properties of *Peganum harmala* L. in traditional Iranian medicine and modern phytotherapy: a review. *Journal of Traditional Chinese Medicine*.

[4] Sheahan M. C., Chase M. W. (1996). A phylogenetic analysis of Zygophyllaceae R. Br. based on morphological, anatomical and rbcL sequence data. *Botanical Journal of the Linnean Society*.

[5] Decreane L. P. R., Delact J., Smets E. F. (1996). Morphological studies in Zygophyllaceae. II. The floral development and vascular anatomy of *Peganum harmala*. *American Journal of Botany*.

[6] Kartal M., Altun M. L., Kurucu S. (2003). HPLC method for the analysis of harmol, harmalol, harmine and harmaline in the seeds of *Peganum harmala* L.. *Journal of Pharmaceutical and Biomedical Analysis*.

[7] Abbott L. B., Gregory T., Bettmann T., Sterling R. M. (2008). Physiology and recovery of African rue (*Peganum harmala*) seedlings under water-deficit stress. *Weed Science*.

[8] Zhao T., Wang Z. T., Branford-White C. J., Xu H., Wang C. H. (2011). Classification and differentiation of the genus *Peganum* indigenous to China based on chloroplast trnL-F and psbA-trnH sequences and seed coat morphology. *Plant Biology (Stuttgart, Germany)*.

[9] Aslam N., Wani A. A., Nawchoo I. A., Bhat M. A. (2014). Distribution and medicinal importance of *Peganum harmala*—a review. *International Journal of Advanced Research*.

[10] Asgarpanah J., Ramezanloo F. (2012). Chemistry, pharmacology and medicinal properties of *Peganum harmala* L.. *African Journal of Pharmacy and Pharmacology*.

[11] Wang Z., Wan H., Li J., Zhang H., Tian M. (2013). Molecular imaging in traditional Chinese medicine therapy for neurological diseases. *BioMed Research International*.

[12] Karasawa M. M. G., Mohan C. (2018). Fruits as prospective reserves of bioactive compounds: a review. *Natural products and bioprospecting*.

[13] Amartuvshin N., Shagdar D., Tserenbaljid G. (2006). Taxonomy of the genus *Peganum* in Mongolia. *Mongolian Journal of Biological Sciences*.

[14] El Bahri L., Chemli R. (1991). *Peganum harmala* L.: a poisonous plant of North Africa. *Veterinary and Human Toxicology*.

[15] Ehsanpour A. A., Saadat E. (2002). Plant regeneration from hypocotyl culture of *Peganum harmala*. *Pakistan Journal of Botany*.

[16] Mahmoudian M., Jalilpour H., Salehian P. (2002). Toxicity of *Peganum harmala*: review and a case report. *Iranian Journal of Pharmacology and Therapeutics*.

[17] Abbott L. B., Lepak D., David L. D. (2007). Vegetative and reproductive phenology of African rue (*Peganum harmala*) in the Northern Chihuahuan Desert. *The South Western Naturalist.*.

[18] Farouk L., Laroubi A., Aboufatima R., Benharref A., Chait A. (2008). Evaluation of the analgesic effect of alkaloid extract of *Peganum harmala* L.: possible mechanisms involved. *Journal of Ethnopharmacology*.

[19] Yousefi R., Ghaffarifar F., Dalimi A. (2009). The effect of *Alkanna tincturia* and *Peganum harmala* extracts on *Leishmania major* in vitro. *Iranian Journal of Parasitology*.

[20] Duran A., Hamzaoğlu E. (2002). Flora of Kazankaya Canyon (Yozgat-Corum). *Turkish Journal of Botany*.

[21] AS S., Kudrina N. O., Kulmanov T. E., Kurmanbayeva M. S., Inelova Z. A., Shalgimbayeva S. M. (2019). Anatomical and morphological structure of *Peganum harmala* of Almaty region and its therapeutic properties. *Pakistan Journal of Botany*.

[22] Narantsetseg A., Shagdar D., Tserenbaljid G. (2006). Taxonomy of the genus *Peganum L* (Peganaceae Van Tieghem) in Mongolia. *Mongolian Journal of Biological Sciences*.

[23] Khan N. A., Raina A., Wagay N. A., Tantray Y. R. (2017). Distribution, status, pharmacological, and traditional importance of *Peganum harmala* L.. *International Journal of Advanced Research in Science, Engineering*.

[24] Grubov V. I. (1982). Opredeliteli sosudistikh rastenii Mongolii, *Nauka*. *Leningradskoe otdelenie*.

[25] United States Department of Agriculture (USDA) (2008). *Peganum harmala: Agricultural Research Service (ARS)*.

[26] Prain D., Bishen S., Mahendra P. S., Dehra D. (2010). *Bengal Plants*.

[27] Grubov V. I. (1998). Conspectus of Zygophyllaceae R. Br in Central Asia. *News of Vascular Plants*.

[28] Bobrov E. G. (2004). Zygophyllaceae. *Flora of USSR*.

[29] Moloudizargari M., Mikaili P., Aghajanshakeri S., Asghari M. H., Shayegh J. (2013). Pharmacological and therapeutic effects of *Peganum harmala* and its main alkaloids. *Pharmacognosy Reviews*.

[30] Upadhyay B., Roy S., Kumar A. (2007). Traditional uses of medicinal plants among the rural communities of Churu district in the Thar Desert, India. *Journal of ethnopharmacology.*.

[31] Tahraoui A., El-Hilaly J., Israili Z. H., Lyoussi B. (2007). Ethnopharmacological survey of plants used in the traditional treatment of hypertension and diabetes in south-eastern Morocco (Errachidia province). *Journal of Ethnopharmacology*.

[32] Lansky E. S., Lansky S., Paavilainen H. M. (2017). *Harmal: The Genus Peganum*.

[33] Pieroni A., Muenz H., Akbulut M., Başer K. H., Durmuşkahya C. (2005). Traditional phytotherapy and trans-cultural pharmacy among Turkish migrants living in Cologne, Germany. *Journal of ethnopharmacology*.

[34] Leporatti M. L., Ghedira K. (2009). Comparative analysis of medicinal plants used in traditional medicine in Italy and Tunisia. *Journal of Ethnobiology and Ethnomedicine*.

[35] Nedelcheva A., Dogan Y., Obratov-Petkovic D., Padure I. M. (2011). The traditional use of plants for handicrafts in southeastern Europe. *Human Ecology*.

[36] Ma Z. Z., Hano Y., Nomura T., Chen Y. J. (2000). Three new triterpenoids from *Peganum nigellastrum*. *Journal of Natural Products*.

[37] Apostolico I., Aliberti L., Caputo L. (2016). Chemical composition, antibacterial and phytotoxic activities of *Peganum harmala* seed essential oils from five different localities in Northern Africa. *Molecules*.

[38] Salehi B., Selamoglu Z., Sener B. (2019). *Berberis* plants-drifting from farm to food applications, phytotherapy, and phytopharmacology. *Foods*.

[39] Tahrouch S., Rapior S., Belahsen Y., Bessière J.-M., Andary C. (1998). Volatile constituents of *Peganum harmala* (Zygophyllaceae). *Acta Botanica Gallica*.

[40] Faridi P., Ghasemi Y., Mohagheghzadeh A. (2013). Chemical composition of *Peganum harmala* smoke and volatile oil. *Journal of Essential Oil-Bearing Plants*.

[41] Selim S. A., Aziz M. H. A., Mashait M. S., Warrad M. F. (2013). Antibacterial activities, chemical constitutes and acute toxicity of Egyptian *Origanum majorana* L., *Peganum harmala* L. and *Salvia officinalis* L. essential oils. *African Journal of Pharmacy and Pharmacology*.

[42] Amariz I. A., da Silva J. P., Pereira E. C. V. (2019). Chemical study of *Peganum harmala* seeds. *African Journal of Biotechnology*.

[43] Benzekri R., Bouslama L., Papetti A., Hammami M., Smaoui A., Limam F. (2018). Anti HSV-2 activity of *Peganum harmala* (L.) and isolation of the active compound. *Microbial Pathogenesis*.

[44] Li Y., He Q., Du S., Guo S., Geng Z., Deng Z. (2018). Study of methanol extracts from different parts of *Peganum harmala* L. using 1H-NMR plant metabolomics. *Journal of analytical methods in chemistry*.

[45] Wang Z., Kang D., Jia X. (2018). Analysis of alkaloids from *Peganum harmala* L. sequential extracts by liquid chromatography coupled to ion mobility spectrometry. *Journal of Chromatography B*.

[46] Wang K. B., Hu X., Li S. G. (2018). Racemic indole alkaloids from the seeds of *Peganum harmala*. *Fitoterapia*.

[47] Moazeni M., Ardakani Z. S., Saharkhiz M. J. (2017). In vitro ovicidal activity of *Peganum harmala* seeds extract on the eggs of *Fasciola hepatica*. *Journal of Parasitic Diseases*.

[48] Wang K. B., Li D. H., Bao Y. (2017). Structurally diverse alkaloids from the seeds of *Peganum harmala*. *Journal of Natural Products*.

[49] Yang Y. D., Cheng X. M., Liu W. (2016). Peganumine B-I and two enantiomers: new alkaloids from the seeds of *Peganum harmala* Linn. and their potential cytotoxicity and cholinesterase inhibitory activities. *RSC Advances*.

[50] Wang C., Zhang Z., Wang Y., He X. (2016). Cytotoxic constituents and mechanism from *Peganum harmala*. *Chemistry & Biodiversity*.

[51] Wang K. B., Li D. H., Hu P. (2016). A series of *β*-carboline alkaloids from the seeds of *Peganum harmala* show G-quadruplex interactions. *Organic Letters*.

[52] Chabir N., Ibrahim H., Romdhane M. (2015). Seeds of *Peganum harmala* L. chemical analysis, antimalarial and antioxidant activities, and cytotoxicity against human breast cancer cells. *Medicinal Chemistry*.

[53] Tascon M., Benavente F., Sanz-Nebot V. M., Gagliardi L. G. (2015). Fast determination of harmala alkaloids in edible algae by capillary electrophoresis mass spectrometry. *Analytical and Bioanalytical Chemistry*.

[54] Sassoui D., Seridi R., Azin K., Usai M. (2015). Evaluation of phytochemical constituents by GC-MS and antidepressant activity of *Peganum harmala* L. seeds extract. *Asian Pacific Journal of Tropical Disease*.

[55] Yang Y., Cheng X., Liu W., Chou G., Wang Z., Wang C. (2015). Potent AChE and BChE inhibitors isolated from seeds of *Peganum harmala* Linn by a bioassay-guided fractionation. *Journal of Ethnopharmacology*.

[56] Wang C. H., Zeng H., Wang Y. H. (2015). Antitumor quinazoline alkaloids from the seeds of *Peganum harmala*. *Journal of Asian Natural Products Research*.

[57] Wang C., Zhang Z., Wang Y., He X. (2015). Cytotoxic indole alkaloids against human leukemia cell lines from the toxic plant *Peganum harmala*. *Toxins.*.

[58] Yang Y. D. (2014). *Cholinesterase Inhibitive Activity-Guided Isolation of Chemical Constituents from Peganum harmala Seeds*.

[59] Shan M., Ma G. Z., Xea L. (2014). Selection of methyl esterification for quantitative analysis of fatty acids from seeds of *Peganum harmala*. *Journal of Chinese Medicinal Materials*.

[60] Wang K. B., Di YT B. Y., Yuan C. M. (2014). Peganumine A, a *β*-carboline dimer with a new octacyclic scaffold from *Peganum harmala*. *Organic Letters*.

[61] Wang K. B., Yuan C. M., Xue C. M. (2014). Pegaharmalines A and B, two novel *β*-carboline alkaloids with unprecedented carbon skeletons from *Peganum harmala*. *RSC Advances*.

[62] Khan F. A., Maalik A., Iqbal Z., Malik I. (2013). Recent pharmacological developments in *β*-carboline alkaloid “Harmaline”. *European Journal of Pharmacology*.

[63] Lamchouri F., Toufik H., Elmalki Z. (2013). Quantitative structure–activity relationship of antitumor and neurotoxic *β*-carbolines alkaloids: nine harmine derivatives. *Research on Chemical Intermediates*.

[64] Liu L., Zhao T., Cheng X., Wang C., Wang Z. (2013). Characterization and determination of trace alkaloids in seeds extracts from *Peganum harmala* Linn. using LC-ESI-MS and HPLC. *Acta Chromatographica*.

[65] Im J. H., Jin Y. R., Lee J. J. (2009). Antiplatelet activity of *β*-carboline alkaloids from *Perganum harmala*: a possible mechanism through inhibiting PLC*γ*2 phosphorylation. *Vascular Pharmacology*.

[66] Zheng X., Zhang Z., Chou G. (2009). Acetylcholinesterase inhibitive activity-guided isolation of two new alkaloids from seeds of *Peganum nigellastrum* Bunge by an in vitro TLC- bioautographic assay. *Archives of Pharmacal Research*.

[67] Frison G., Favretto D., Zancanaro F., Fazzin G., Ferrara S. D. (2008). A case of *β*-carboline alkaloid intoxication following ingestion of *Peganum harmala* seed extract. *Forensic Science International*.

[68] Pulpati H., Biradar Y. S., Rajani M. (2008). High-performance thin-layer chromatography densitometric method for the quantification of harmine, harmaline, vasicine, and vasicinone in *Peganum harmala*. *Journal of AOAC International*.

[69] Li Y. K. (2005). *Study on the Chemical Constituents of Above Ground of Peganum harmala L*.

[70] Shahverdi A. R., Monsef-Esfahani H. R., Nickavar B., Bitarafan L., Khodaee S., Khoshakhlagh N. (2005). Antimicrobial activity and main chemical composition of two smoke condensates from *Peganum harmala* seeds. *Zeitschrift für Naturforschung. Section C*.

[71] Sharaf M., El-Ansari M. A., Matlin S. A., Saleh N. A. (1997). Four flavonoid glycosides from *Peganum harmala*. *Phytochemistry*.

[72] Fan Z. R., Yao X. S. (1992). Studies on the constituents and pharmacological effects of *Peganum*. *Journal Shenyang College of Pharmacy*.

[73] Ma J., Wang X., Zhao S. (1997). Amino acid components in leaves of *Peganum* and the relationship with species' resistance to adverse environment. *Journal of Desert Research*.

[74] Zhu Y. P., Fei Z., Liu M. C., Jia F. C., Wu A. X. (2012). Direct one-pot synthesis of Luotonin F and analogues via rational logical design. *Organic Letters*.

[75] Liu W. (2016). *Studies on the Mechanism of Improving Learning and Memory and the Effect of Antitussive, Bronchodilating of Aerial Parts of Peganum Harmala and Its Active Constituents*.

[76] Ayoob I., Hazari Y. M., Lone S. H., Khuroo M. A., Fazili K. M., Bhat K. A. (2017). Phytochemical and cytotoxic evaluation of *Peganum harmala*: structure activity relationship studies of harmine. *ChemistrySelect*.

[77] Iranshahy M., Fazli Bazaz S., Haririzadeh G., Abootorabi B. Z., Mohamadi A. M. (2019). Chemical composition and antibacterial properties of *Peganum harmala* L.. *Avicenna Journal of Phytomedicine.*.

[78] Moussa T. A., Almaghrabi O. A. (2016). Fatty acid constituents of *Peganum harmala* plant using gas chromatography-mass spectroscopy. *Saudi Journal of Biological Sciences.*.

[79] Li Y., He Q., Geng Z., Du S., Deng Z., Hasi E. (2018). NMR-based metabolomic profiling of Peganum harmala L. reveals dynamic variations between different growth stages. *Royal Society open science*.

[80] Zhenle C., Zhibin L., Jinao D., Ronghan Z., Shouxun Z. (1994). Studies on the volatile constituents of *Peganum* three plants in China. *Journal China Pharmaceutical University.*.

[81] Yang F., Chen R., Feng L., Li H., Zhang H., Liang J. (2010). Chemical constituents from the aerial part of *Peganum nigellastrum*. *Chinese Journal of Natural Medicines*.

[82] Movafeghi A., Abedini M., Fathiazad F., Aliasgharpour M., Omidi Y. (2009). Floral nectar composition of *Peganum harmala* L.. *Natural Product Research*.

[83] Javzan S., Selenge D., Amartuvshin N., Nedelcheva D., Christov V., Philipov S. (2015). Alkaloids from Mongolian species of *Peganum multisectum* (Maxim) Bobrov. *Mongolian Journal of Chemistry*.

[84] Liu W., Cheng X., Wang Z., Wang C. (2013). Vasicine: research advances in resources, pharmacological activities, pharmacokinetics, toxicity and analysis methods. *Journal of Pharmaceutical Research International*.

[85] Duan J., Zhou R., Zhao S., Wang M., Che C. (1998). Studies on the chemical constituents of *Peganum multisectum* Maxim. I. The alkaloids from seeds and antitumour activity. *Journal of China Pharmaceutical University*.

[86] Yang F., Feng L., Li H. D., Zhang H., Chen R. (2010). A new flavone glycoside from the aerial part of *Peganum nigellastrum*. *Chemistry of Natural Compounds*.

[87] Ma Z. Z., Hano Y., Nomura T., Chen Y. J. (2000). Alkaloids and phenylpropanoids from *Peganum nigellastrum*. *Phytochemistry*.

[88] Hashemi Sheikh Shabani S., Seyed Hasan Tehrani S., Rabiei Z., Tahmasebi Enferadi S., Vannozzi G. P. (2015). *Peganum harmala* L.’s anti-growth effect on a breast cancer cell line. *Biotechnology Reports*.

[89] Ivanova A., Serly J., Christov V., Stamboliyska B., Molnar J. (2011). Alkaloids derived from genus *Veratrum* and *Peganum* of Mongolian origin as multidrug resistance inhibitors of cancer cells. *Fitoterapia*.

[90] Lamchouri F., Settaf A., Cherrah Y. (2000). In vitro cell-toxicity of *Peganum harmala* alkaloids on cancerous cell-lines. *Fitoterapia*.

[91] Bournine L., Bensalem S., Fatmi S. (2017). Evaluation of the cytotoxic and cytostatic activities of alkaloid extracts from different parts of *Peganum harmala* L. (Zygophyllaceae). *European Journal of Integrative Medicine.*.

[92] Zhao T., Ding K. M., Zhang L., Cheng X. M., Wang C. H., Wang Z. T. (2013). Acetylcholinesterase and butyrylcholinesterase inhibitory activities of *β*-carboline and quinoline alkaloids derivatives from the plants of genus *Peganum*. *Journal of Chemistry*.

[93] Azadniya E., Morlock G. E. (2019). Automated piezoelectric spraying of biological and enzymatic assays for effect-directed analysis of planar chromatograms. *Journal of Chromatography. A*.

[94] Deng G., Liu W., Ma C. (2019). *In vivo* and *in vitro* metabolism and pharmacokinetics of cholinesterase inhibitor deoxyvasicine from aerial parts of *Peganum harmala* Linn in rats via UPLC-ESI-QTOF-MS and UPLC-ESI-MS/MS. *Journal of Ethnopharmacology*.

[95] Moradi M. T., Karimi A., Fotouhi F., Kheiri S., Torabi A. (2017). *In vitro* and *in vivo* effects of *Peganum harmala* L. seeds extract against influenza A virus. *Avicenna journal of phytomedicine*.

[96] Soliman A. M., Abu-El-Zahab H. S., Alswiai G. A. (2013). Efficacy evaluation of the protein isolated from *Peganum harmala* seeds as an antioxidant in liver of rats. *Asian Pacific Journal of Tropical Medicine*.

[97] Herraiz T., González D., Ancín-Azpilicueta C., Arán V. J., Guillén H. (2010). *β*-Carboline alkaloids in *Peganum harmala* and inhibition of human monoamine oxidase (MAO). *Food and Chemical Toxicology*.

[98] Jiang B., Li S., Liu W. (2015). Inhibitive activities detection of monoamine oxidases (MAO) A and B inhibitors in human liver MAO incubations by UPLC-ESI-MS/MS. *Journal of Pharmaceutical and Biomedical Analysis*.

[99] Jiang B., Meng L., Zou N. (2019). Mechanism-based pharmacokinetics-pharmacodynamics studies of harmine and harmaline on neurotransmitters regulatory effects in healthy rats: challenge on monoamine oxidase and acetylcholinesterase inhibition. *Phytomedicine*.

[100] Fatma B., Fatiha M., Elattafia B., Noureddine D. (2016). Phytochemical and antimicrobial study of the seeds and leaves of *Peganum harmala* L. against urinary tract infection pathogens. *Asian Pacific Journal of Tropical Disease.*.

[101] Nenaah G. (2010). Antibacterial and antifungal activities of *β*-carboline alkaloids of *Peganum harmala* (L) seeds and their combination effects. *Fitoterapia*.

[102] Abolhassanzadeh Z., Aflaki E., Yousefi G., Mohagheghzadeh A. (2015). Randomized clinical trial of *Peganum* oil for knee osteoarthritis. *Journal of Evidence-Based Complementary & Alternative Medicine*.

[103] Liu W., Cheng X., Wang Y. (2015). *In vivo* evaluation of the antitussive, expectorant and bronchodilating effects of extract and fractions from aerial parts of *Peganum harmala* Linn. *Journal of Ethnopharmacology*.

[104] Liu W., Wang Y., He D. D. (2015). Antitussive, expectorant, and bronchodilating effects of quinazoline alkaloids (±)-vasicine, deoxyvasicine, and (±)-vasicinone from aerial parts of *Peganum harmala* L.. *Phytomedicine*.

[105] Singh V. K., Mishra V., Tiwari S. (2013). Anti-secretory and cyto-protective effects of peganine hydrochloride isolated from the seeds of *Peganum harmala* on gastric ulcers. *Phytomedicine*.

[106] Mennenga S. E., Gerson J. E., Dunckley T., Bimonte-Nelson H. A. (2015). Harmine treatment enhances short-term memory in old rats: dissociation of cognition and the ability to perform the procedural requirements of maze testing. *Physiology & Behavior*.

[107] He D., Wu H., Wei Y. (2015). Effects of harmine, an acetylcholinesterase inhibitor, on spatial learning and memory of APP/PS1 transgenic mice and scopolamine-induced memory impairment mice. *European Journal of Pharmacology*.

[108] Fortunato J. J., Réus G. Z., Kirsch T. R. (2009). Acute harmine administration induces antidepressive-like effects and increases BDNF levels in the rat hippocampus. *Progress in Neuro-Psychopharmacology & Biological Psychiatry*.

[109] Fortunato J. J., Réus G. Z., Kirsch T. R. (2010). Effects of *β*-carboline harmine on behavioral and physiological parameters observed in the chronic mild stress model: further evidence of antidepressant properties. *Brain Research Bulletin*.

[110] Fortunato J. J., Réus G. Z., Kirsch T. R., Stringari R. B., Fries G. R. (2010). Chronic administration of harmine elicits antidepressant-like effects and increases BDNF levels in rat hippocampus. *Journal of Neural Transmission*.

[111] Liu W., Zhu Y., Wang Y. (2017). Anti-amnesic effect of extract and alkaloid fraction from aerial parts of *Peganum harmala* on scopolamine-induced memory deficits in mice. *Journal of Ethnopharmacology*.

[112] El-Saad E.-R. M. (1980). *Peganum harmala*: its use in certain dermatoses. *International Journal of Dermatology*.

[113] Yazdanbod A., Sadeghifard N., Niknam M. (2001). Therapeutic effects, adverse events and spinal-Z pharmaceutical indicators in the treatment of cancer of the upper gastrointestinal tract. *Ardebil Univeristy of Medical Sciences*.

[114] Mashak B., Hoseinzadeh M., Ehsanpour A., Ghanbaran A. R., Vakili M. (2017). Evaluation of treatment response and side effects of spinal-Z in patients with metastatic gastroesophageal adenocarcinoma: a double-blind randomized controlled trial. *Jundishapur Journal of Chronic Disease Care*.

[115] Panahi Y., Saadat A., Seifi M., Rajaee M., Butler A. E., Sahebkar A. (2018). Effects of spinal-Z in patients with gastroesophageal cancer. *Journal of pharmacopuncture*.

[116] Shirani-Boroujeni M., Heidari-Soureshjani S., Keivani H. Z. (2017). Impact of oral capsule of *Peganum harmala* on alleviating urinary symptoms in men with benign prostatic hyperplasia; a randomized clinical trial. *Journal of Renal Injury Prevention*.

[117] Shaheen H. A., Issa M. Y. (2020). _*In vitro*_ and _*in vivo*_ activity of _*Peganum harmala*_ L. alkaloids against phytopathogenic bacteria. *Scientia Horticulturae*.

[118] Rezzagui A., Merghem M., Derafa I., Dahamna S. (2020). Acute and sub-acute toxic effects of Algerian *Peganum harmala* L. crud extract. *Journal of Drug Delivery and Therapeutics.*.

